# Supporting parents of preschool children in adopting a healthy lifestyle

**DOI:** 10.1186/1472-6955-11-12

**Published:** 2012-08-01

**Authors:** Lucie Lemelin, Frances Gallagher, Jeannie Haggerty

**Affiliations:** 1 Nursing Department, UQO, Saint-Jérôme, Canada; 2 Nursing School, Sherbrooke University, Sherbrooke, Canada; 3 Epidemiology Department, McGill University, Montreal, Canada

**Keywords:** Overweight, Childhood, Preschool, Parental opinion, Health promotion, Action research

## Abstract

**Background:**

Childhood obesity is a public health epidemic. In Canada 21.5% of children aged 2–5 are overweight, with psychological and physical consequences for the child and economic consequences for society. Parents often do not view their children as overweight. One way to prevent overweight is to adopt a healthy lifestyle (HL). Nurses with direct access to young families could assess overweight and support parents in adopting HL. But what is the best way to support them if they do not view their child as overweight? A better understanding of parents’ representation of children’s overweight might guide the development of solutions tailored to their needs.

**Methods/design:**

This study uses an action research design, a participatory approach mobilizing all stakeholders around a problem to be solved. The general objective is to identify, with nurses working with families, ways to promote HL among parents of preschoolers. Specific objectives are to: 1) describe the prevalence of overweight in preschoolers at vaccination time; 2) describe the representation of overweight and HL, as reported by preschoolers’ parents; 3) explore the views of nurses working with young families regarding possible solutions that could become a clinical tool to promote HL; and 4) try to identify a direction concerning the proposed strategies that could be used by nurses working with this population. First, an epidemiological study will be conducted in vaccination clinics: 288 4–5-year-olds will be weighed and measured. Next, semi-structured interviews will be conducted with 20 parents to describe their representation of HL and their child’s weight. Based on the results from these two steps, by means of a focus group nurses will identify possible strategies to the problem. Finally, focus groups of parents, then nurses and finally experts will give their opinions of these strategies in order to find a direction for these strategies. Descriptive and correlational statistical analyses will be done on the quantitative survey data using SPSS. Qualitative data will be analyzed using Huberman and Miles’ (2003) approach. NVivo will be used for the analysis and data management.

**Discussion:**

The anticipated benefits of this rigorous approach will be to identify and develop potential intervention strategies in partnership with preschoolers’ parents and produce a clinical tool reflecting the views of parents and nurses working with preschoolers’ parents.

## Introduction

The focus of this study is the promotion of a healthy lifestyle from an early age to prevent increasing overweight in preschool children. This proposal first outlines the background, the study goals and objectives, and the literature review. The literature concerns overweight in preschool children and associated factors, parents’ perception of their child’s weight and current efforts to promote a healthy lifestyle in young children. The proposal then describes the research design and setting, the sampling, data collection and analysis strategies. Finally, ethical considerations and anticipated benefits are discussed.

## Background

Childhood obesity is a public health epidemic; 22 million children under the age of 5 are affected [1]. In Canada 21.5% of children aged 2–5 years [2] and 13.5% of four-year-olds in Quebec (Canada) are overweight [3]. The prevalence doubled between 1978 and 2005. Though self-reported data suggests a more recent stabilization in prevalence, Lamontagne and Hamel [4] caution against over-confidence in this trend because of the social-desirability bias of self-reported data. Although many factors are involved, lifestyle is at the heart of the problem. In the first years of life, parents play a leading role in the development of a healthy lifestyle [5-7].

Lifestyle and thus modifiable factors associated with overweight in preschool children include frequent consumption of sweetened beverages [8], high-calorie processed foods [9], dietary restrictions [10,11], hours of sleep [12], physical inactivity, and hours spent in front of a screen [13]. So it is not surprising that prevention efforts focus on adopting a healthy lifestyle, namely a healthy diet and increased physical activity [14].

In 2006 Quebec adopted a six-year action plan to promote healthy lifestyles and prevent weight-related problems in children [6]. Priorities include promoting a healthy diet and physically active lifestyle. The plan is aimed at various authorities, including daycare and early childhood educational services, but no direct action with families is envisaged nor have strategies to promote healthy lifestyles in families been defined. One of the objectives of the Quebec program “Integrated perinatal and early childhood services for families living in vulnerable situations” is to improve family lifestyles. However, this program is aimed at a specific group and not the entire population [15]. The vulnerable target population presents health risks such as substance abuse, alcoholism and violence.

The preschool period is a good time to act. This is confirmed by longitudinal studies on the growth curve. The aim is to delay the adiposity rebound^a^ since if it occurs early (before age 6), the risk of being overweight later increases [16-18].^a^The results of a study by Nader et al. [16] indicate that if a child is overweight at age 5, i.e. has a BMI >85^th^ percentile, he/she is five times more likely to be overweight at age 12. However, normal changes in body form and structure at this age [19], complicate accurate perception of overweight.

Being overweight has serious consequences: children can develop low self-esteem and be socially isolated [20] and have a poor self-image as young as age 5 [21]. They also are at risk of cardiovascular disease, modification of the lipid profile, sleep apnea, hypertension and type 2 diabetes in adulthood [22]. Economically, obesity costs the Canadian health care system about $4.3 billion in 2000–2001 [23].

Despite the consequences of overweight for the child and for society, few parents view their children as overweight [24,25]. Few studies have attempted to do an in-depth analysis of the reasons for this erroneous view. The representation of childhood overweight and a healthy lifestyle from the parents’ point of view, which has received little or no attention to date, could shed light on the reasons for this misperception. Representation consists of beliefs, attitudes, opinions and knowledge about something [26]. Thus it guides the actions of parents and, in this case, behaviours and lifestyles of children.

In Quebec, nurses regularly see children for vaccinations and other services prior to school entry. These contacts present an opportunity to take action in early childhood. But how can nurses support families in adopting healthy habits? To answer this question, we have designed an action research study involving nurses, parents and experts to identify the views of parents and healthcare professionals regarding the promotion of a healthy lifestyle and to develop clinical tools based on new knowledge derived from the description of the parents’ representation.

### Relevance of the study

Currently, in the absence of systematic interventions to promote a healthy lifestyle among parents of preschoolers, it is imperative for nurses to understand the parents’ point of view in order to provide them with adequate support. The relevance of this study is that it is important to understand the representation of overweight and healthy lifestyles among preschoolers’ parents in order to intervene early and meet their needs effectively. This study is justified because these elements must be clarified in order to tailor interventions to promote healthy lifestyles accordingly. Moreover, the development of healthy lifestyles, healthy development in childhood and the health services available are three determinants of health that need to be targeted to prevent overweight in Canada [7]. Developing a clinical tool for public health nurses will facilitate the provision of evidence-based services and is likely to improve the quality of services. Professional practices will be relevant and based on the viewpoint of parents as key players in adopting healthy lifestyles for their children and helping to address overweight, which is a serious public health problem.

## Study goals and objectives

The aim of the study is to identify, with nurses working with families, possible solutions to promote healthy lifestyles among parents of preschool children after exploring parents’ representation of overweight and healthy lifestyles in preschoolers. The specific objectives are to: 1) document the current situation (estimate the prevalence of overweight and describe the associated factors) in preschool children attending a vaccination clinic in a region of Quebec, Canada, 2) describe the representation of overweight and health lifestyles according to parents of preschoolers, 3) explore the viewpoint of nurses working with young families concerning possible interventions that could be used to develop a clinical tool to promote healthy lifestyles, and 4) achieve a direction on which strategies to promote and which of the proposed strategies to prioritize.

## Literature review

We present current knowledge concerning the issue of overweight in preschool children, specifically: the etiology of overweight and known associated factors, the importance of acting in early childhood and the parents’ perception and representation of this issue. Finally, current health promotion efforts to address overweight in children and the conceptual framework used for this research will be presented and justified.

### Etiology of overweight and associated factors

For the purpose of this study, the term ‘overweight’ includes both excess weight and obesity, which are measured by body mass index (BMI). ^b^In adults, a BMI of 25 to 30 indicates overweight, and over 30, obesity. In children, three BMI cut-points have been proposed by CDC [27], WHO [28] and IOTF [29], respectively. For the CDC, BMI percentiles classify children as overweight (BMI >85^th^) and obese (BMI ≥95^th^). The WHO uses the z-score of BMI-for-age, with children classified as overweight with a BMI between one and two standard deviations (SD) above the mean and as obese with more than two SD above the mean. This translates to overweight as a BMI >84^th^ and obese as a BMI >97.7^th^ percentile. To obtain accurate baseline values, the IOTF defined BMI thresholds that take the child’s age and sex into account. For IOTF, a child is considered overweight with a BMI ≥91th and obese with a BMI ≥99^th^ percentile for their age-sex referent. The IOTF criteria constitute an international definition of childhood overweight. The reference values are based on data obtained from six different countries. This definition makes it possible to assess the prevalence of overweight and compare it to other studies because it was developed specifically for international comparaison.

Etiologically, overweight occurs when calorie intake—the amount of energy consumed–exceeds caloric expenditure over a long period of time [30,31]. In North America, restaurant portions for children are 24% larger than necessary [32], convenience food is high in calories [33] and children consume large amounts of sweetened beverages [8,34]. The availability of ready-to-eat food in grocery stores and ‘fast food’ restaurants contribute to changes in families’ eating habits [9]. As for energy expenditure, a sedentary lifestyle, time spent in front of a screen [13,35,36] and the presence of a screen (television or monitor) in the child’s bedroom all contribute to weight gain [37].

Other parental factors increase the risk of the child being overweight: a) one or both parents being overweight leads to a 50% or 70% higher risk respectively [38]; b) low family income makes it difficult to afford healthy food [39]; c) mother not having completed high school [13]; and d) dietary restrictions (forbidden fruit is always more attractive) [10,11]. Also, the risk is higher in preschool children who sleep less than 10 hours per night [12].

### Acting early in childhood to promote healthy lifestyles

As mentioned earlier, if the adiposity rebound occurs before age 6, the risk of later obesity is high compared to an adiposity rebound occurring after age 6 [16-18]. Because of this, it is important to act early. In a study by Canning, Courage and Frizzell [40] involving 4161 children from three to five years old, the results show that it is important to take action as young as age 3 since one in four children is overweight by the age of 3½. Knowing that young children can be influenced by the food environment of the adults around them, this factor must be considered when considering the best time to intervene [41]. When the child is young, the family is the place to develop healthy behaviors [5], hence the importance of supporting parents in adopting healthy lifestyles when their children are very young.

### Parents’ perception of their child’s weight

Quantitative studies on perception identified in the literature aim to determine if parents are aware of their child’s weight. Six studies, published in English or French, concerning preschool children were published between 2003 and 2010 are presented. Table1 summarizes the main findings of these studies.

**Table 1:** Parental perception of child’s weight

**Authors/Country**	**Design/Aim**	**n/yrs**	**Results**
Bossink-Tuna et al. (2009) [24]	Cross-sectional surveys	635	87% of parents of overweight children disagreed that their child was overweight
Netherlands		2–4	
Carnell et al. (2005)	Determine parents’ perceptions of their child’s weight compared to measured weight	564	98.1% of parents of overweight children and 82.9% of parents of obese children did not perceive him/her as overweight
United Kingdom [25]		3–5	
Manios et al. (2009)		2287	55.4% of parents of overweight children and 88.0% of at-risk-of-overweight children perceived him/her as normal weight
Greece [42]		2–5	
Maynard et al. (2003)		5500	32.1% of mothers of overweight children reported that he/she was about the right weight
United States [43]		2–11	
Vuorela et al. (2010)		310	72.7% of parents of overweight boys and 93.8% of overweight girls classified them as normal weight
Finland [44]		5	
Wald et al. (2007)		612	82.5% of the parent of 3–5 years-old children their overweight children did not identify their child as overweight
United States [45]		3–12	

Despite differences in the children’s age, target populations and slight variations in how weight perceptions were measured, all of the studies found that the parents did not view their overweight child as having excess weight. The study by Maynard, Galuska, Blanck and Serdula [43] and Manios et al. [42] are the only two with a representative sample of the population nationally. The other studies shown in Table1 looked at samples from a program for underprivileged children in which overweight is more pronounced. Only four of the five studies concerned preschoolers only.

### Parents’ representation of overweight and lifestyle

Parents’ representation of overweight could partly explain why they do not see it, which could in turn guide efforts to promote healthy lifestyles. Many authors have studied the concept of representation [46-52]. Representation is a mental activity by which a group reconstitutes the reality before it and attributes a meaning to it [46]. Abric [26] proposes an operational definition that is the sum of opinions, attitudes, beliefs and knowledge about a specific situation. Also, the representation guides behavior and is a precursor to taking action [46].

There are no studies on parents’ representation of overweight and healthy lifestyles in preschool children; there are a few studies on the representation of food. However, Pelicand and Doumont [53] identified 50 exclusively or partly qualitative studies on the perception of the child’s weight to shed some light, from interview transcripts, on representation. Among the factors identified was a belief that obesity is less important than other health risks like passive smoking [54] and that overweight is a medical problem when the child cannot participate in physical activities with peers [55]. Moreover, according to some parents, overweight children are “big and strong” and a “big baby” is a sign of good health and good parenting [43]. The younger the child, the more overweight is made light of by mothers, believing that the child will inevitably grow and overweight will disappear [55]. Other parents think that weight and height are preprogrammed and thus impossible to change [55]. These aspects are interesting but cannot be used to comprehensively identify parents’ representation of overweight and healthy lifestyles in children. A description of opinions, attitudes, beliefs and knowledge, i.e. a comprehensive representation of overweight and healthy lifestyles, could help to understand why parents see little or no overweight when it is present in their child. This knowledge could also help guide current efforts to promote healthy lifestyles in parents of young children.

### Interventions to promote healthy lifestyles and prevent overweight in children

The aim of the meta-analysis by Flynn et al. [56] was to identify programs advocating obesity prevention best practices. A total of 147 programs were found and 26% were considered feasible, useful and effective. The best practices identified were those targeting the school population and including a BMI assessment, identification of chronic disease risk factors and participants’ involvement in physical activities. One of the findings was a lack of programs for children up to age 5 and widely-varying practices. A limitation of this meta-analysis was the broad inclusion criteria for programs making them difficult to compare in terms of effectiveness because of their different designs and measures. Summerbell et al. [57] also did a meta-analysis of 22 randomized studies to measure the impact of interventions addressing diet or physical activity or a combination of the two in children. The results show that the studies on interventions are rarely conclusive and that it is difficult to compare results because of differing designs, interventions and measures. Of the studies identified by these two meta-analyses, two specifically targeted preschool children and give an insight into possible overweight prevention interventions.

The first study by Harvey, Berino and Rouke [58] was a randomized clinical trial with 43 mothers of two-year-olds aimed at determining the effectiveness of helping immigrant parents to reduce obesity. In-home meetings provided education on diet and physical activity. The focus for the intervention was to improve parenting skills to develop appropriate eating and exercise behaviors to prevent obesity. The results showed a 0.27 decline in the mothers’ BMI and a commitment to monitor their child’s food intake. In this study, the randomization of the sample was not specified, only mothers with a BMI ≥25 were included and the majority of the mothers were Native American, which limits the generalizability of the results. In the second study by Dennison, Russo, Burdick and Jenkins [59] involving 83 mother/child dyads, the aim was to develop and evaluate an intervention to reduce television viewing time. The intervention programme consisted of 39 weeks of 1-hour sessions, including 32 sessions on healthy eating and seven sessions to encourage reduction of TV viewing for both parents and children. The stratified randomized clinical trial showed that the children reduced their television viewing time but actual times were not measured; parental estimates of the child’s sedentary activity in previous week were lower than before the intervention. Knowing that the time spent in front of a screen reduces the time spent on physical activities, the results seem interesting. However, the impact on weight was not presented. In summary, the results of intervention studies are inconclusive and few of them looked at preschool children.

Despite none or minimal impact on weight, these studies demonstrate short and medium-term changes in health habits, notably in better food choices, increased physical activity and less sedentary behavior. Identifying appropriate strategies and interventions for successful health promotion requires a precise definition of the problem, identification of the target group (parents and professionals), and the influencing factors (home environment) [60].

Thus, the evidence of interventions to reduce childhood overweight is inconclusive. With this study, we hope to establish a foundation for designing a future intervention based on the ecologic model, that will also take into account the evidences actually published. To that end, we are taking into consideration the points of view of the major determinant of the child environment, the parent. Understanding what influences the parent’s representation of overweight will help design interventions adapted to the stages of change. We also target clinicians who come into predictable and regular encounters with the parent during the preschool period. Exploring the viewpoint and engaging clinicians and local decision-makers will outline the scope of influence and action they can exert, especially in targeting actions or communication that take into account parental representation of overweight. Finally, we will consider experts opinion on the subject to encompass a broader scope of potential strategies to better support parents in promoting healthy lifestyles for their children.

### Conceptual framework

In view of the findings regarding parents’ misperception of overweight in their preschool children, the fundamental role that parents play in the adoption of children’s healthy lifestyles and interventions that primarily target a change in behavior by the adoption of healthy lifestyles, the conceptual framework used for this study is the Transtheoretical Model [61]. This is a model used to change behaviors [62]. According to the Transtheoretical Model, behavioral change takes place through five stages, allowing us to understand when changes in thoughts, intentions, attitudes or behaviors occur [63]. The stages in the model are outlined in Table2. This model has also been used in interventions with families. For example, Weiss [64] based the therapeutic education of asthmatic children and their families on this model. 

**Table 2:** Stages of the transtheoretical model

	**Description**	**Interventions**
**1**	Does not feel the need for change, denies the problem, has no interest in change	Evaluate self-confidence, discuss the pros and cons of the behavior to be changed
**2**	Recognizes the problem, gathers information, does not take action	Raise awareness
**3**	Decides to act, plans the change	Discuss the pros and cons, help and guide the individual in his/her approach, remind of past successes
**4**	Applies new behavioral strategies	Discuss the pros and cons of the change and provide positive reinforcement
**5**	Incorporates changes in own lifestyle	Positively reinforce desired behaviors, remind of successes and recognize danger signs
**6**	Initial problem is no longer a temptation	

From the literature review, it appears that interventions promoting healthy lifestyles with parents of young children are more likely to reach a population aware of the overweight issue (stages 3 and 4) because they see a problem that concerns them. The interventions, aimed at adopting a healthy diet or doing physical activity, correspond to changes in behavior that require first an intention to act with regard to the problem perceived as important and representing a definite risk for the child. If parents do not view their child as being overweight, why would they intend to change their lifestyle? Thus there is an incompatibility between the stage many parents are at and the stage targeted by interventions. Given that most parents are at stages 1 and 2 with respect to their children’s overweight, it is vital to understand parents’ representation of overweight and healthy lifestyles in preschool children in order to develop strategies to promote healthy lifestyles and raise awareness (stages 1 and 2) by clarifying parents’ beliefs, attitudes, opinions and knowledge.

Nonetheless, promotion of health lifestyles much take into account the environment in which parents and their children adopt health behaviours. Thus, the ecologic model of Bronfenbrenner (1979) will be used as the conceptual background in order to consider the different elements to be considered in the design of interventions to promote adoption of healthy behaviours. So, the Transtheoretical Model will allow health professionals to adapt interventions to the stage of change and of motivation of the parent, integrating both parents’ representation of child overweight and knowledge coming from the literature. The ecological model will allow intervention on obesogenic environment which parents sometimes inadvertently create.

## Method

### Research design

The action research design is a systematic and participatory investigation approach based on the pragmatic-interpretative paradigm [65,66]. This design, through cycles or loops, helps to understand problems and formulate actions directed towards problem-solving [67]. The ultimate aim, objectives and orientations are based on discussion and negotiation with the participants. The researcher takes a participatory attitude to achieve real cooperation [67]. The type of action research proposed by Stringer and Genat [67] targets reflexive professional practice aimed at empowering professional groups to defend clients’ interests. Thus the focus is on the professional who defines the problem based on his/her own practice. The change targets professional practice and the solutions emerging from the professionals and target population. In this study, nurses working with preschool children try to determine possible interventions to promote healthy lifestyles among parents of preschool children to prevent increasing overweight.

In the present action research four cycles (loops) are proposed. Each loop meets a study objective and is composed of a reflection phase and an action phase. Each loop will be described in terms of the methodological approach, sampling strategy and data collection and analysis methods. First, however, the research setting is described.

### Research setting

For this study the research team consists of two professors and a doctoral student. Throughout the study, an advisory committee of stakeholders in the region where the study is conducted will contribute at various steps in the study. The committee members will monitor the study and participate in the thinking regarding its objectives. They come from the local health and social services agency, network of daycare centres, CSSS, the municipality and parents.

The intervention area is a region in the province of Quebec, Canada. The population of this region was 43,953 in 2008 and included 6006 children up to 14 years of age [68]. The preschool vaccination clinics reach many families in both urban and rural areas.

### Awareness of overweight in preschoolers (loop 1)

As the starting point for the study, the nurses participating in the study want to know more about overweight in their preschool population. The first step will be to conduct a prevalence survey. This will provide the participating nurses and researchers with a profile of the population served to initiate thinking and discussions of the study issue. Descriptive quantitative data will be collected to document the situation by estimating the prevalence of overweight and describe the known risk factors.

#### Sample (loop 1)

The study population will be composed of preschoolers who came to vaccination clinics in 2010. The probabilistic sample will comprise 288 consecutive children, for a 95% confidence level, a 5% margin of error and a prevalence that could go up to 25%. The 25% estimate will provide sufficient statistical power. The sample will be created with the consent of the nurses working in the vaccination clinics.

#### Data collection (loop 1)

The variable of interest is childhood overweight (BMI) for which the reference values used will be those developed by Cole et al. [29]. The following variables will be described: the child’s age, sex, health problems and medications, the family’s postal code (socioeconomic level), the parents’ education, and the height and weight (self-declared) of the parent bringing the child to the vaccination clinic. Data will be collected with a self-administered questionnaire developed specifically for the study, which will be cognitively tested with ten parents to ensure the clarity of the questions, visual presentation and time required to complete it. It will be anonymous and a consent form will be given and explained to parents prior to the questionnaire. At the vaccination clinic, the student-researcher will measure the child’s height and weight and enter this information on the questionnaire, together with the age and sex. She will then give the questionnaire to the parent. At this point, the parent can decide to complete the questionnaire or not. It is expected to take five minutes. A subsection of the questionnaire will invite the parent to voluntarily provide contact information to participate in an interview or focus group in the later loops in the study. After completion, the questionnaire will be left at a specified place.

### Description of parents’ representation of overweight and lifestyle (loop 2)

For loops 2, 3 and 4, an inductive qualitative approach will be used to explore the views of parents, nurses, and experts. The qualitative approach has the advantage of describing in depth important social aspects relating to a situation [69]. This approach will also encourage nurses to participate in the search for intervention strategies to promote healthy lifestyles adapted to both the parents’ needs and the situation at the clinic. Throughout these loops, the researcher will keep a log documenting the views of the student researcher and the effect of the site on the student.

Loop 2 will start with presenting to the participant nurses the results obtained in loop 1, namely the population profile. Then discussions will take place between the nurses and student researcher concerning the information required from parents to continue the reflection. The objective of loop 2 is to describe the representation of parents of preschool children of overweight and healthy lifestyles of children in this age group. The representation will be constructed from parents’ beliefs, opinions, attitudes, knowledge and perceptions about their preschool child’s weight and life habits. This information will give the nurses and researchers a better understanding of the parents’ views and help them reflect on possible strategies to facilitate the promotion of healthy lifestyles with parents of preschool children.

#### Sample (loop 2)

The study population is composed of parents of four- and five-year-olds who come to the preschool vaccination clinic. The purposive sample will be created on the basis of the BMI calculated from Cole et al.’s [29]reference values. The sample will include 20 parents (key informants) of a normal weight or overweight child who, on the questionnaire completed in the previous step, agreed to be contacted for the next step in the study. The inclusion criteria will be having completed the questionnaire in loop 1 and understanding French. Parents of children with health problems (e.g. Trisomy 21) or taking medication that can affect weight (e.g. corticosteroids) will be excluded from this part of the study. Taking into account factors associated with overweight identified in loop 1 will ensure maximum variability when selecting the loop 2 participants.

#### Data collection (loop 2)

Data will be collected in two semi-structured interviews, conducted with the help of an evolving guide. The interviews will explore the concept of the representation of overweight and lifestyles from the parents’ viewpoint. The first will last about 60 minutes and the second 30 minutes. For the interviews, the researchers have developed an interview guide based on the literature on representation, overweight and healthy lifestyles. The guide will be added to, improved and modified following the discussions with the advisory committee and nurses during the presentation of the results from loop 1 and in the light of findings from the literature. A few examples of questions in the guide are as follows: What words or images come to mind when you think about the lifestyles and weight of four- and five-year-old children? Talk to me about children’s weight and lifestyles. Four- and five-year-old children can be overweight—What do you think about that? What challenges do parents face concerning lifestyles?

The procedure will consist of a first telephone call to the parents to explain the objective of this loop, answer their questions, obtain their consent and make an appointment. The consent form will be read and signed before the first interview. The second in-depth interview will take place three to five weeks after the first and serves to specify the researchers’ understanding of the first interview and to explore in-depth some additional issues. A summary of the previous interview will be sent to the parent before the second interview. All interviews will be recorded and transcribed. Data will be considered saturated when there is data redundancy and no new information is identified [69,70].

### Search for possible solutions to promote healthy lifestyles (loop 3)

The objective of this loop is to explore the views of nurses working with young families concerning possible strategies to develop a clinical tool to promote healthy lifestyles based on the results from the previous loop. The search for interventions will be affected by the results obtained concerning the parents’ views on the representation of overweight and healthy lifestyles, the conceptual framework (transtheoretical model) and relevant literature.

#### Sample (loop 3)

The sample will consist of 9 participating nurses working with the target population. As participants, the nurses and researchers will work together to identify possible interventions adapted to the population’s needs.

#### Data collection (loop 3)

The data will be collected through a focus group discussion, using a guide designed for this purpose. The aim of the questions will be to document the nurses’ views of the results from loop 2. First, a meeting will be held with the nurses to present the objective of this third loop and give them a consent form. They will be given a week to complete and return the consent form by mail to the student researcher. The focus group is an approach that has a flexible structure, is quick and inexpensive and is aimed at developing a consensus. The focus group will be held with a facilitator and an observer. The facilitator will ask questions, encourage participation [71] and manage the order of speaking and dominant members [72]. The observer will take notes, verify the content discussed and clarify certain elements. The meeting will last 2 hours and the discussion will be recorded and transcribed.

### Search for direction concerning the proposed strategies (loop 4)

The objective of this last loop is to guide the choice of potential solutions stemming from the work done in the previous loop with the nurses. The solutions will be discussed with the nurses themselves, parents and experts.

#### Sample (loop 4)

Three purposive groups will be formed. The first group will consist of six parents of preschool children, the second group will involve nine participating nurses working with the target population, and the third will consist of six advisors who have agreed to act as experts (a pediatrician, nutritionist, nurse, community health worker, endocrinologist, other researchers)

#### Data collection (loop 4)

This loop involves three phases. First, a focus group discussion with seven parents will obtain their views regarding the proposed strategies. Second, a focus group discussion with ten nurses will gather obtain their views regarding the proposed solutions. Both focus groups will be held with a facilitator and an observer. They will last 60 to 90 minutes and the discussions will be recorded and transcribed. Informed consent will be obtained from the participants (parents and nurses) before the meetings. From both groups we will identify strategies, privileging those that emerge independently from both groups and those that the nurses are confident they can implement. Third, a postal questionnaire including open-ended questions will be sent to the seven experts asking for their opinion regarding the possible strategies suggested by the parents, nurses and researchers. The experts will be requested to rank the suggested strategies by expected impact and relevance. This loop with identify a small number of strategies that will be form the starting point of a subsequent intervention study.

### Data analysis (loops 1, 2, 3 and 4)

The statistical analyses of the data from loop 1 will provide a prevalence estimate, i.e., percentage of children of normal and overweight (excess weight and obese). SPSS software will be used.

For qualitative data, recorded sessions will be transcribed and analyzed using Huberman & Miles [73] method. For loop 2, parental representation, the data will be condensed using a preliminary analysis grid with various categories, based on predetermined categories that emerged from the literature review, to which emerging categories will be added. The data will be regrouped according to common characteristics of elements in the content and the four representation attributes (beliefs, attitudes, opinions and knowledge). Two researchers will code the data, which will be presented using matrices based on conceptual groupings. The analysis process will start by listening to the interviews, then coding the content of the interviews. The analysis grid will be refined throughout the process because the data from loop 2 will be analyzed concurrently with the interviews. Finally after reviewing the categories and coding and discussions with the advisory committee, the determination of a final categorization can be completed. The same process will be used for focus group discussions in loops 3 and 4. During the analysis process, there will be regular discussions with the research team. Then, based on the data analyzed, literature review and views of the experts, strategies to promote healthy lifestyles (solutions considered) can be proposed. The qualitative data will be managed with NVivo software.

#### Rigor of the research process

Throughout the action research, rigorous methodological standards will be met. Credibility, which is the correspondence between the participants’ constructions and the reconstructions formulated, will be achieved by an iterative process between researchers, parents and nurses. In addition, triangulation of data sources (parents, professionals, experts and literature) and triangulation of researchers will ensure better correspondence between the results and field data. Data will be co-analyzed including a reflexive stance and there will be frequent team discussions. Finally, recording and transcribing the individual interviews and focus groups will make it possible to confirm the findings by independent analysis. The context, participants’ characteristics and results will be detailed in depth, which facilitates transferability. To ensure reliability, i.e., taking into account the evolution of the phenomenon, a lot of time will be spent in the field and discussions with the advisory committee will be held throughout the study [69]. A logbook will be kept and an observer will attend the focus groups in order to keep track of the contextual aspects.

## Ethical considerations

The study was approved by the research ethics boards of the Centre hospitalier universitaire de Sherbrooke (CHUS) and Université de Sherbrooke and the Centre de Santé et des Services sociaux (CSSS) Rivière-du-Nord/Nord de Mirabel. Parents and nurses will be asked to sign free and informed consent forms before each data collection. Parents and nurses will be able to withdraw from the study at any time without prejudice. Anonymity will be preserved for those parents who do not wish to be contacted again, and no nominative information will be retained. Confidentiality will be safeguarded in the usual way, such as by keeping the data under lock and key for 5 years, with access restricted to the research team.

The main ethical issues in this study relate to stigmatization of the child and culpability of the parent. Preschool children are a vulnerable group. It will not be possible to identify them (the questionnaire in loop 1 does not ask for children’s names), which reduces the risk of being stigmatized. Also, to reduce the risk of stigmatization, we will ask the parents for permission to weigh and measure all the children, regardless of their apparent weight, for the study. The risk of stigmatization arises later in the lives of children who are overweight for a significant period of time. The parents of children of both normal and overweight will be interviewed, which will minimize the culpability aspect potentially related to the subject of the study. Precautions will be taken when formulating the questions for the interview guide used in loop 2. In addition, psychological support for the family will be available if required at the local health centre (a partner in the study). Particular attention will be paid throughout the study so that parents feel competent in their role.

## Anticipated benefits

The results will give an appreciation of the magnitude of the weight problem in a specific region of Québec and the opportunity to evaluate weight in a vaccination clinic, will describe parents’ representation of overweight and healthy lifestyles in preschoolers, will initiate reflection for the professionals involved and will put in place actions designed to equip parents and families of preschool children in order to prevent and reduce the risks of being overweight. Identification by the professionals of strategies to promote healthy lifestyles in families with preschoolers will be derived from evidence-based data from parents and the literature. The professionals involved in this action research will become more aware of the phenomenon and more expect to benefit from a transfer of knowledge. In addition, the nurses will have resources that will help them intervene and make maximum use of meetings with parents of preschool children to address this health problem. The proposed solutions will foster the integration of new interventions into current practice and open the door to future research on these interventions.

## Conclusion

This action research will create partnerships between professionals, parents and experts; these, in turn, are expected to lead to interventions to promote the adoption of healthy lifestyles and prevent overweight in young children. The specific nature of the proposed interventions will be co-determined with the different stakeholders. Specifically, depending on the orientations that emerge from the current research, we will use participatory methods to combine input from our different stakeholders with the evidentiary base on interventions to design an intervention. Again drawing on participatory and qualitative methods, we will test the acceptability and feasibility of the proposed intervention to broader sample of parents and health professionals. We will follow with a pilot study, then a full experimental design to evaluate its efficacy. In addition, the partner and members of the advisory committee for this study will work closely with the researchers to suggest innovative ways to disseminate the results and to integrate results into practice.

## Endnotes

^a^The adiposity rebound is the increase in body mass index (BMI) after age 6: the BMI declines until age 6 as height increases faster than weight. At age 6 height and weight increase simultaneously and the curve rebounds.

^b^BMI = Weight (kg) / Height (m)^2^.

## Competing interests

The authors declare that they have no competing interests

## Authors’ contributions

The protocol was designed and drafted by Lucie Lemelin in the context of her doctoral studies in Nursing Sciences. Frances Gallaher, as principal supervisor, and Jeannie Haggerty as co-supervisor, advised on design and provided input to various versions of the protocol. All authors read and approved the final manuscript.

## Pre-publication history

The pre-publication history for this paper can be accessed here:

http://www.biomedcentral.com/1472-6955/11/12/prepub
